# Parental knowledge, vaccine hesitancy, and practices regarding seasonal influenza vaccination for preschool-aged children in Shenzhen, China: Insights from a cross-sectional survey

**DOI:** 10.1371/journal.pone.0353478

**Published:** 2026-07-09

**Authors:** Wu Li, Hui-ling Tan, Chun-yan Zhuang, Jun-yu Li, Xu Xie, Gang Li

**Affiliations:** 1 Longgang District Center for Disease Control and Prevention, Shenzhen, China; 2 Longcheng Subdistrict Public Health Service Center, Longgang District, Shenzhen, China; 3 Shenzhen Center for Disease Control and Prevention, Shenzhen, China; CAMS PUMS IMI: Chinese Academy of Medical Sciences & Peking Union Medical College Institute of Medical Information, CHINA

## Abstract

**Background:**

Seasonal influenza poses a substantial health threat to children under 5 years of age. Despite recommendations for annual seasonal influenza vaccination (SIV), coverage among preschool-aged children in China remains low. Vaccine hesitancy is an important barrier to vaccine uptake. This study assessed parental knowledge, vaccine hesitancy, and practices regarding SIV for preschool-aged children in Shenzhen, China, and identified factors associated with hesitancy.

**Methods:**

A cross-sectional online survey was conducted from September to December 2025 using a multistage sampling strategy across six administrative districts of Shenzhen. Parents of children aged 0–5 years were recruited from community health service centers and kindergartens/childcare institutions. An adapted Chinese version of the Parental Attitudes toward Childhood Vaccines (PACV) scale was used to assess hesitancy toward SIV. Parents’ knowledge regarding influenza and SIV, as well as SIV practices, were assessed and scored. Independent factors associated with hesitancy scores were identified using multivariable linear regression, and factors associated with binary hesitancy outcomes were conducted using multivariable logistic regression. Sensitivity analyses excluding the behavioral PACV items were also performed.

**Results:**

A total of 17,955 parents were included in the final analysis, and 19.16% reported that their child had received SIV in the past year. The mean PACV hesitancy score was 32.26 ± 18.50, and 19.42% of parents (3,487/17,955) were classified as vaccine hesitant. The highest proportions of hesitant responses were related to concerns about serious adverse reactions (69.47%), the safety of repeated annual vaccination (55.61%), and doubts regarding vaccine effectiveness (52.68%). Higher knowledge and vaccination practice scores were independently associated with lower levels of vaccine hesitancy in both multivariable linear and logistic regression analyses (both *P* < 0.001). Additionally, older parental age, Shenzhen household registration, employment in enterprises/public institutions, middle-level household income, kindergarten attendance, obtaining vaccine information from kindergartens and relatives/friends were negatively associated with vaccine hesitancy in multivariable logistic regression analyses (all *P* < 0.05). Sensitivity analyses yielded generally consistent findings.

**Conclusion:**

Parental hesitancy toward SIV among preschool-aged children in Shenzhen was low-to-moderate and was associated with knowledge, prior vaccination behaviors, and sociodemographic characteristics. Concerns about vaccine safety and effectiveness were the most frequently cited reasons for hesitancy. Targeted communication strategies delivered through multiple channels, together with consideration of free SIV for preschool-aged children may help improve vaccine uptake in this high-burden population.

## 1. Introduction

Seasonal influenza is a highly prevalent acute respiratory infection that poses a substantial public health threat, particularly to children under 5 years of age [1,2]. Owing to their immature immune systems, young children experience higher infection rates, greater susceptibility to severe complications, and higher risks of hospitalization than adults, resulting in a disproportionate disease burden [1–4]. In 2018, an estimated 109.5 million influenza episodes and 10.1 million influenza-associated acute lower respiratory infection cases occurred globally among children younger than 5 years, leading to approximately 34,800 acute lower respiratory infection-related deaths, most of which occurred in low- and lower-middle-income countries [2]. These findings underscore the need for effective public health interventions to reduce influenza-related morbidity and mortality in early childhood.

Seasonal influenza vaccination (SIV) is one of the most cost-effective strategies for preventing influenza and mitigating its consequences. Annual vaccination is recommended for all individuals aged ≥ 6 months without contraindications [3,5]. Evidence shows that SIV in children reduces the incidence of influenza illness [6], decreases secondary transmission within households and communities [7,8], and lowers the risks of hospitalization and severe outcomes [9,10]. Despite these benefits, substantial global variation remains in parental acceptance and uptake of childhood SIV [11].

In China, SIV is not included in the National Immunization Program (NIP) and is generally offered on a voluntary, self-paid basis. National influenza vaccination coverage remains low, with estimated overall uptake rates of 3.16% and 2.47% during the 2020–2021 and 2021–2022 influenza seasons, respectively [12]. A national cross-sectional study reported an overall SIV coverage of only 2.4% among adults aged ≥40 years during the 2014–2015 influenza season, with 1.7% in those aged 40–59 years and 3.8% in those aged ≥60 years [13]. Among children younger than 5 years, SIV coverage remains suboptimal and shows substantial regional variation. For example, SIV coverages among preschool-aged children has been estimated at 2.9% in Beijing [14], 7.4% in Fuzhou [15], and 22.5% in Shanghai [16], which are markedly lower than that reported in many high-income settings [17–19]. Although recent national guidelines have increasingly emphasized childhood influenza vaccination to reduce disease burden [20], a substantial gap remains between recommendations and real-world uptake.

Vaccine hesitancy – defined as motivational state of being conflicted about, or opposed to, getting vaccinated, includes intentions and willingness – has emerged as a critical barrier to achieving optimal coverage [21]. Previous studies have identified multiple sociodemographic and psychosocial determinants of parental hesitancy toward childhood SIV, including child-related factors, parental characteristics, socioeconomic status, and perceptions of vaccine benefits, barriers, susceptibility, and severity [11]. Given regional differences in socioeconomic conditions and population composition, context-specific determinants of parental hesitancy toward childhood SIV warrant further investigation.

Shenzhen, a megacity in southern China with a registered population exceeding 23 million in 2024 and a gross domestic product of more than 3.68 trillion RMB, has implemented progressive influenza vaccination policies for many years. Since 2016, the Shenzhen municipal government has provided free SIV to adults aged ≥ 60 years, and since 2019, free influenza vaccination has also been provided to primary and secondary school students, achieving relatively high coverage (about 60%) and favorable public health impact [22,23]. However, preschool-aged children, who bear a substantial influenza burden, have not been included in the free vaccination program. Moreover, local evidence on SIV coverage, parental vaccine hesitancy, and related determinants among preschool-aged children in Shenzhen remains limited. To inform evidence-based policy making, we conducted a cross-sectional survey among parents of preschool-aged children in Shenzhen to assess their knowledge of influenza and influenza vaccination, levels of vaccine hesitancy, and vaccination practices. The findings may help optimize local influenza immunization strategies and inform consideration of publicly funded vaccination for this high-risk population.

## 2. Methods

### 2.1. Survey design and participants

A cross-sectional survey was conducted from September to December 2025 in Shenzhen, China. The minimum sample size was calculated using the standard formula for estimating a single population proportion: $N={Z_{\frac{\alpha }{\text{2}}}}^{\text{2}}\times p\times (\text{1}-p)/d^{\text{2}}$. A parental influenza vaccine hesitancy prevalence (p) of 37.11% was assumed based on a previous survey investigating parental hesitancy toward SIV among children aged <18 years in Longhua District, Shenzhen [24]. Although the previous study used a Health Belief Model framework rather than the PACV scale, it investigated a similar population and research topic and therefore provided the most relevant local estimate available at the time of study design. Using a permissible error of 0.01, and a 95% confidence level, the minimum sample size was estimated at 8,966. After adjustment for a design effect of 1.5 and a 10% non-response rate, the final minimum required sample size was 14,794.

A multistage sampling strategy was adopted. Six administrative districts of Shenzhen (Futian, Nanshan, Longgang, Bao’an, Luohu, and Longhua) were included to capture major urban, economic, and demographic settings within the city. According to 2024 public statistical data [25], these districts accounted for approximately 87.7% of Shenzhen’s gross domestic product, 87.3% of its permanent resident population, and 65.0% of its land area. Therefore, although the study did not include all administrative districts of Shenzhen, the selected districts covered most of the city’s population and economic activity and represented diverse urban contexts within Shenzhen.

Within each selected district, three community health service centers and three kindergartens/childcare institutions were selected by simple random sampling. Community health service centers were included because they are the main providers of routine childhood vaccination services in Shenzhen, whereas kindergartens and childcare institutions were included because they provide direct access to parents of preschool-aged children. Eligible participants were parents of preschool-aged children (0–5 years) whose children either attended the selected kindergartens/childcare institutions or visited the selected community health service centers during the study period. The minimum number of participants recruited from each district was proportionally allocated according to the distribution of the registered resident population. The inclusion criteria were as follows: (1) father or mother of a child aged 0–5 years; (2) residence in Shenzhen for at least 6 months; (3) provision of informed consent; and (4) ability to understand and complete the questionnaire independently. Participation was voluntary, and informed consent was obtained from all participants. Parents’ decisions to participate or withdraw at any stage did not affect their children’s entitlement to routine services at childcare institutions or medical services at community health service centers. The study was approved by the Ethics Committee of Shenzhen Longgang District Center for Disease Control and Prevention (Approval No. LGCDC2025003).

### 2.2. Survey content

The questionnaire comprised four domains. (1) Sociodemographic characteristics: information was collected on parents (e.g., age, sex, ethnicity, education, occupation, household registration, income, and marital status) and children (e.g., age, sex, health status, and kindergarten attendance). (2) Knowledge of seasonal influenza and SIV: six items assessed knowledge of the differences between influenza and the common cold, disease severity, prevention measures, the recommended age for vaccination, the need for annual vaccination, and the optimal vaccination period. Each correct response was scored as 1 and each incorrect response as 0, yielding a total score of 0–6; scores were categorized as 0–2, 3–4, and 5–6. (3) Vaccination practices: six items assessed parental and child vaccination behaviors, including any parental vaccination in the past year, parental influenza vaccination in the past year, timely childhood NIP vaccination, uptake of non-NIP vaccines, previous child influenza vaccination, and child influenza vaccination in the past year. Each affirmative response was scored as 1, yielding a total score of 0–6; scores were categorized as 0–2, 3–4, and 5–6. (4) Parental vaccine hesitancy: hesitancy was assessed using an adapted Chinese version of the Parental Attitudes toward Childhood Vaccines (PACV) scale [26,27], modified to address SIV specifically. The adapted scale consisted of 15 items across three domains: vaccination behavior (2 items), vaccine safety and effectiveness (4 items), and general attitudes (9 items). Item scoring followed the standard PACV framework, with scores of 2, 1, and 0 indicating hesitancy, uncertainty, and non-hesitancy, respectively. For items 1 and 2, responses of “not sure” were treated as missing values with no imputation performed, as these responses could not be clearly classified as hesitant or non-hesitant attitudes in the adapted Chinese version. Questionnaires with more than two missing items were considered invalid and excluded from the analysis. To account for occasional missing values, the raw total score was linearly transformed to a 0–100 scale using the following formula: $\text{transformed score}=\;\frac{Raw\;total\;score}{\text{2}\times number\;of\;completed\;items}\times \text{1}\text{0}\text{0}\;$. This approach standardizes the PACV score according to the number of completed items and minimizes bias caused by limited missing data. Consistent with the original PACV scoring approach and previous validation studies [27,28], participants with transformed scores ≥50 were classified as vaccine hesitant, whereas those with scores <50 were classified as non-hesitant.

### 2.3. Survey procedures and quality control

Before formal data collection, a pilot survey was conducted to refine questionnaire wording and ensure clarity. Data were collected through an online questionnaire hosted on the Wenjuanxing platform (Changsha Ranxing Information Technology Co., Ltd., China). Participants accessed the survey by scanning a QR code displayed at the study sites. Before accessing the questionnaire, all potential participants were required to read an online informed consent form on the first page of the survey. The consent form explained the study purpose, survey procedures, voluntary nature of participation, anonymity, confidentiality, potential risks and benefits, and the right to withdraw at any time. Only parents who clicked the “agree” button were allowed to proceed to the questionnaire; those who did not provide consent could not access the survey. The electronic consent process was automatically documented by the Wenjuanxing platform.

To ensure data quality, questionnaires were excluded if the completion time was less than 5 minutes or if logically inconsistent responses were identified. Each mobile device was allowed to submit the questionnaire only once. After submission, participants were redirected to a health education page; because this occurred after questionnaire completion, it did not influence survey responses.

### 2.4. Statistical analysis

Data were analyzed using IBM SPSS Statistics version 23.0. Continuous variables with an approximately normal distribution were summarized as mean ± standard deviation (SD), and categorical variables were summarized as frequencies and percentages. Internal consistency reliability of the PACV scale was assessed using Cronbach’s α, and construct validity was assessed using the Kaiser-Meyer-Olkin (KMO) measure. The distribution of PACV scores was evaluated using histograms and Q-Q plots. Group comparisons were performed using independent-samples t tests or one-way analysis of variance, as appropriate, with Bonferroni correction for post hoc comparisons when necessary. The assumptions of linear regression, including residual normality and homoscedasticity, were assessed using residual plots. Variables with *P* < 0.20 in univariate analyses were entered into a multivariable regression model using the enter method to identify factors independently associated with hesitancy scores. Multicollinearity was assessed using variance inflation factors, with values below 5 considered acceptable. In addition to treating the transformed PACV score as a continuous outcome, vaccine hesitancy was also analyzed as a binary outcome (score ≥50 were classified as vaccine hesitant, whereas those with a score <50 were classified as non-hesitant). Multivariable logistic regression was performed using the same candidate variables as those included in the multivariable linear regression model. Regression coefficients (β), adjusted odds ratios (aORs) and 95% confidence intervals (CIs) were reported. As a sensitivity analysis, the two behavioral items (Q1 and Q2) were removed from the PACV scale, and the multivariable linear regression analyses were repeated using revised PACV scores based only on the safety/efficacy and general attitudes domains. All tests were two-sided, and *P* < 0.05 was considered statistically significant.

## 3. Results

### 3.1. Enrollment of study participants and sociodemographic characteristics

A total of 20,031 individuals completed the online questionnaire. Among them, 19,320 provided informed consent and agreed to participate, yielding a participation rate of 96.45%. We excluded 1,038 respondents whose completion time was less than 5 minutes and 327 respondents with logically inconsistent answers. Ultimately, 17,955 participants were included in the final analysis. Of the included participants, 1,750 (9.75%) were from Futian District, 2,009 (11.19%) from Nanshan District, 4,523 (25.19%) from Longgang District, 5,136 (28.60%) from Bao’an District, 1,324 (7.37%) from Luohu District, and 3,213 (17.90%) from Longhua District. The mean age of participants was 35.42 ± 5.40 years. Fathers accounted for 2,825 (15.73%) respondents. In addition, 9,834 (54.77%) participants had Shenzhen household registration, 17,201 (95.80%) were of Han ethnicity, 17,482 (97.37%) were married, and 12,547 (69.88%) had attained a bachelor’s degree or above.

### 3.2. Parental responses to the PACV scale

The overall Cronbach’s α coefficient of the PACV scale was 0.81, and the KMO measure was 0.85, indicating good internal consistency and sampling adequacy. The Cronbach’s α coefficients for the three domains-vaccination behavior, vaccine safety and effectiveness, and general attitudes-were 0.61, 0.69, and 0.78, respectively, with corresponding KMO values of 0.62, 0.70, and 0.86.

Based on the linearly transformed PACV total score (0–100 scale), the mean parental hesitancy score toward SIV for preschool-aged children was 32.26 ± 18.50, and the overall prevalence of vaccine hesitancy (score ≥ 50) was 19.42% (3,487/17,955). The mean hesitancy scores for the three domains-vaccination behavior, vaccine safety and effectiveness, and general attitudes were 2.60 ± 4.31, 17.24 ± 7.98, and 12.42 ± 12.16, respectively; the differences among domains were statistically significant (*F* = 13,045.50, *P* < 0.001).

At the item level, the proportion of hesitancy ranged from 12.24% to 26.15% in the vaccination behavior domain, 29.61% to 69.47% in the vaccine safety and effectiveness domain, and 0.76% to 28.27% in the general attitudes domain. Among the 15 items, Q13 (trust in information from official authorities) and Q14 (ability to communicate concerns with healthcare providers) had the lowest hesitancy proportions, at 1.32% and 0.76%, respectively. In contrast, Q4 (concern about serious adverse reactions after vaccination), Q5 (concern about the safety of repeated vaccination during childhood) Q6 (concern about the effectiveness of SIV) had the highest hesitancy proportions, at 69.47%, 55.61% and 52.68%, respectively (Table 1).

**Table 1:** Parental responses to the Parental Attitudes toward Childhood Vaccines (PACV) scale in Shenzhen, China.

Scale dimensions and items	Scoring scheme for response options	No. of hesitant cases	%
**Vaccination behavior**			
Q1. Excluding illness or allergy, have you ever postponed seasonal influenza vaccination for your child for other reasons?	Yes = 2 points, No = 0 points, and Not sure was treated as missing responses from the total score calculation.	4695	26.15
Q2. Excluding illness or allergy, have you ever declined seasonal influenza vaccination for your child for other reasons?	Yes = 2 points, No = 0 points, and Not sure was treated as missing responses from the total score calculation.	2197	12.24
**Vaccine safety and effectiveness**			
Q3. Do you think it is acceptable for children to receive the seasonal influenza vaccine at the same time as other vaccines?	Strongly agree/agree = 0 points, Uncertain = 1 point, and Disagree/strongly disagree = 2 points.	5316	29.61
Q4. Do you worry about the possibility of serious adverse reactions in your child following seasonal influenza vaccination?	Very worried/somewhat worried = 2 points, Uncertain = 1 point, and not very worried/not worried at all = 0 points.	12473	69.47
Q5. Do you worry that seasonal influenza vaccination may be unsafe every time your child receives it during childhood?	Very worried/somewhat worried = 2 points, Uncertain = 1 point, and not very worried/not worried at all = 0 points.	9985	55.61
Q6. Do you worry that seasonal influenza vaccination may not effectively protect your child against influenza?	Very worried/somewhat worried = 2 points, Uncertain = 1 point, and not very worried/not worried at all = 0 points.	9459	52.68
**General attitudes**			
Q7. To what extent do you agree with having your child receive seasonal influenza vaccination according to the recommended immunization schedule?	0–5 = 2 points, 6–7 = 1 point, and 8–10 = 0 points.	2553	14.22
Q8. Do you think that children today receive more seasonal influenza vaccines than are actually needed?	Strongly agree/agree = 2 points, Uncertain = 1 point, and Disagree/strongly disagree = 0 points.	1121	6.24
Q9. Do you think that receiving the seasonal influenza vaccine can protect against severe influenza or related complications?	Strongly agree/agree = 0 points, Uncertain = 1 point, and Disagree/strongly disagree = 2 points.	672	3.74
Q10. Do you think it is better for children to gain immunity by getting the illness rather than through seasonal influenza vaccination?	Strongly agree/agree = 2 points, Uncertain = 1 point, and Disagree/strongly disagree = 0 points.	3848	21.43
Q11. If you have another child, would you be willing to have them vaccinated against influenza?	Yes = 0 points, No = 2 points, and Uncertain = 1 point.	1542	8.59
Q12. In general, how hesitant are you about vaccinating your child against influenza?	Very hesitant/ somewhat hesitant = 2 points, Uncertain = 1 point, and Not very hesitant/ not hesitant at all = 0 points.	5076	28.27
Q13. Do you trust the information on seasonal influenza vaccination provided by official authorities?	Strongly agree/agree = 0 points, Uncertain = 1 point, and Disagree/strongly disagree = 2 points.	237	1.32
Q14. Can you openly communicate your concerns about your child’s seasonal influenza vaccination with the healthcare provider?	Strongly agree/agree = 0 points, Uncertain = 1 point, and Disagree/strongly disagree = 2 points.	137	0.76
Q15. Overall, how would you rate your level of trust in the healthcare provider administering the seasonal influenza vaccine?	0–5 = 2 points, 6–7 = 1 point, and 8–10 = 0 points.	1738	9.68
Total	The total raw score of the scale was linearly transformed to a 0–100 scale. Scores ≥50 were classified as vaccine hesitancy, whereas scores <50 were classified as non-hesitancy.	3487	19.42

### 3.3. Parental knowledge of seasonal influenza/vaccine and vaccination practices

As shown in Table 2, 83.46% of parents correctly distinguished seasonal influenza from the common cold, 85.73% recognized influenza as a serious health threat to children, and 99.55% were aware of preventive measures against influenza. Most parents (82.66%) knew that SIV is recommended for children aged 6 months or older, and 70.84% knew that annual vaccination is recommended. However, only 64.11% correctly identified September to November as the optimal vaccination period.

**Table 2:** Parental knowledge of seasonal influenza and influenza vaccination in Shenzhen, China.

Questions	Responses	N	%	Hesitancy score (*mean ± SD*)	*t*	*P*
1. Seasonal influenza is distinct from the common cold.	Yes	14986	83.46	30.80 ± 18.23	24.10	<0.001
	No	2969	16.54	39.62 ± 18.08		
2. Seasonal influenza poses severe health hazards to children.	Yes	15392	85.73	30.70 ± 17.89	28.29	<0.001
	No	2563	14.27	41.63 ± 19.34		
3. Awareness of seasonal influenza prevention measures.	Yes	17875	99.55	32.18 ± 18.46	8.56	<0.001
	No	80	0.45	49.90 ± 18.86		
4. Seasonal influenza vaccination is recommended for children aged ≥6 months.	Yes	14841	82.66	30.57 ± 18.20	27.25	<0.001
	No	3114	17.34	40.31 ± 17.77		
5. Annual seasonal influenza vaccination is recommended for children.	Yes	12719	70.84	25.04 ± 13.86	102.65	<0.001
	No	5236	29.16	49.80 ± 16.53		
6. September to November is considered the optimal period for seasonal influenza vaccination.	Yes	11511	64.11	29.15 ± 17.72	30.97	<0.001
	No	6444	35.89	37.83 ± 18.56		

As shown in Table 3, 14.61% of parents reported receiving any vaccine within the past year, and 7.17% specifically reported influenza vaccination. The majority (97.57%) reported ensuring timely vaccination of their children according to the national immunization schedule, 85.49% had previously vaccinated their children with non-NIP vaccines, and 65.29% had previously vaccinated their child against influenza. However, only 19.16% reported that their child had received influenza vaccination in the past year.

**Table 3:** Parental vaccination practices in Shenzhen, China.

Practices	Responses	N	%	Hesitancy score (*mean ± SD*)	*t*	*P*
1. Parental vaccination within the past year.	Yes	2624	14.61	26.38 ± 17.42	17.79	<0.001
	No	15331	85.39	33.27 ± 18.49		
2. Parental influenza vaccination within the past year.	Yes	1288	7.17	20.72 ± 15.31	23.60	<0.001
	No	16667	92.83	33.15 ± 18.42		
3. Parents have ensured timely vaccination of their children according to the national immunization schedule.	Yes	17519	97.57	32.08 ± 18.43	8.27	<0.001
	No	436	2.43	39.48 ± 19.84		
4. Parents have previously vaccinated their children with non–national immunization program vaccines.	Yes	15349	85.49	31.29 ± 18.14	17.16	<0.001
	No	2606	14.51	37.96 ± 19.54		
5. Parents have previously vaccinated their children against influenza.	Yes	11723	65.29	28.53 ± 17.07	38.60	<0.001
	No	6232	34.71	39.29 ± 19.03		
6. Parents have vaccinated their children against influenza within the past year.	Yes	3441	19.16	28.50 ± 17.16	13.32	<0.001
	No	14514	80.84	33.15 ± 18.69		

Across all knowledge items, parents with correct responses consistently had significantly lower hesitancy scores than those with incorrect responses (all *P* < 0.001). Awareness that annual influenza vaccination is recommended was associated with a particularly large difference in mean hesitancy score (25.04 ± 13.86 vs 49.80 ± 16.53, *P* < 0.001). Higher knowledge score categories were associated with progressively lower levels of vaccine hesitancy. Parents with low knowledge scores (0–2) had the highest hesitancy prevalence (55.21%) and mean hesitancy score (50.10 ± 17.23), whereas those with high knowledge scores (5–6) had markedly lower hesitancy prevalence (9.99%) and mean score (26.90 ± 16.09) (*F* = 2120.39, *P* < 0.001; Table 4).

**Table 4:** Characteristics of study participants and univariate analyses of parental vaccine hesitancy.

Characteristics	N (%)	Vaccine hesitancy		Univariable linear regression e	
		N (%)	Scores (*M* ± *SD*)	*t / F*	*P*
Age (years)					
< 35	9624 (53.60)	1896 (19.7)	32.60 ± 18.14	2.64	0.008
≥ 35	8331 (46.40)	1591 (19.1)	31.87 ± 18.90		
Gender					
Mother	15130 (84.27)	2952 (19.51)	32.33 ± 18.39	1.13	0.260
Father	2825 (15.73)	535 (18.94)	31.90 ± 19.07		
Ethnic group					
Han	17201 (95.80)	3328 (19.35)	32.17 ± 18.47	−3.35	0.001
Others ^*a*^	754 (4.20)	159 (21.09)	34.47 ± 19.12		
Marital status					
Married	17482 (97.37)	3370 (19.28)	32.16 ± 18.49	−4.46	<0.001
Others ^*b*^	473 (2.63)	117 (24.74)	36.00 ± 18.63		
Household registration					
Other areas	8121 (45.23)	1634 (20.12)	33.47 ± 17.40	−7.97	<0.001
Shenzhen	9834 (54.77)	1853 (18.84)	31.26 ± 19.30		
Education level					
High school or lower	5408 (30.12)	1154 (21.34)	34.32 ± 17.21	9.82	<0.001
Bachelor’s degree or above	12547 (69.88)	2333 (18.59)	31.37 ± 18.96		
Occupation					
Commercial and service sectors	3460 (19.27)	737 (21.30)	33.61 ± 18.30	30.69	<0.001
Migrant or manual laborers	1169 (6.51)	231 (19.76)	33.11 ± 17.05		
Employees of enterprises or public institutions	9763 (54.37)	1747 (17.89)	31.07 ± 18.61		
Unemployed or others	3563 (19.84)	772 (21.67)	33.96 ± 18.62		
Monthly family income (RMB) ^*c*^					
< 10,000	6120 (34.09)	1282 (20.95)	34.04 ± 17.70	43.22	<0.001
10,000-30,000	7939 (44.21)	1413 (17.80)	31.33 ± 18.30		
≥ 30,000	3896 (21.7)	792 (20.33)	31.36 ± 19.89		
Number of children					
1	13911 (77.48)	2724 (19.58)	32.29 ± 18.63	2.23	0.108
2	3796 (21.14)	710 (18.7)	32.02 ± 18.06		
≥ 3	248 (1.38)	53 (21.37)	34.54 ± 17.43		
Child’s age (years) ^*d*^					
< 3	3069 (17.09)	579 (18.87)	31.84 ± 17.91	−1.39	0.166
3-5	14886 (82.91)	2908 (19.54)	32.35 ± 18.62		
Child’s sex ^*d*^					
Girl	8091 (45.06)	1587 (19.61)	32.00 ± 18.35	−1.72	0.085
Boy	9864 (54.94)	1900 (19.26)	32.48 ± 18.62		
Whether the child attends kindergarten ^*d*^					
No	1010 (5.63)	246 (24.36)	35.29 ± 17.91	5.36	<0.001
Yes	16945 (94.37)	3241 (19.13)	32.08 ± 18.52		
Self-assessment of child health status					
General or weak	1474 (8.21)	313 (21.23)	32.13 ± 19.84	0.29	0.771
Health or good	16481 (91.79)	3174 (19.26)	32.27 ± 18.38		
City of previous vaccination of the child					
Only Shenzhen city	10334 (57.55)	1917 (18.55)	31.56 ± 18.74	20.19	<0.001
Both Shenzhen city and other cities	6599 (36.75)	1361 (20.62)	33.03 ± 18.25		
Only other cities	1022 (5.69)	209 (20.45)	34.44 ± 17.20		
Acquisition of vaccine knowledge from healthcare workers					
No	4795 (26.71)	1039 (21.67)	33.39 ± 19.01	4.92	<0.001
Yes	13160 (73.29)	2448 (18.60)	31.85 ± 18.29		
Acquisition of vaccine knowledge from online media					
No	12338 (68.72)	2571 (20.84)	33.41 ± 18.35	12.42	<0.001
Yes	5617 (31.28)	916 (16.31)	29.73 ± 18.57		
Acquisition of vaccine knowledge from kindergartens					
No	8579 (47.78)	1880 (21.91)	33.18 ± 19.16	6.39	<0.001
Yes	9376 (52.22)	1607 (17.14)	31.42 ± 17.83		
Acquisition of vaccine knowledge from relatives and friends					
No	14886 (82.91)	3029 (20.35)	32.81 ± 18.57	8.70	<0.001
Yes	3069 (17.09)	458 (14.92)	29.62 ± 17.92		
Parents’ knowledge scores on influenza and influenza vaccines					
0-2	1056 (5.89)	583 (55.21)	50.10 ± 17.23	2120.39	<0.001
3-4	4599 (25.61)	1675 (36.42)	42.52 ± 17.90		
5-6	12300 (68.50)	1229 (9.99)	26.90 ± 16.09		
Parents’ practice scores for vaccination					
0-2	5925 (33.00)	1840 (31.05)	39.31 ± 19.01	842.60	<0.001
3-4	10641 (59.26)	1563 (14.69)	29.77 ± 17.16		
5-6	1389 (7.74)	84 (6.05)	21.30 ± 15.68		

Note: Others ^*a*^, ethnic minority groups; Others ^*b*^, unmarried, divorced, or widowed; RMB ^*c*^, Chinese yuan; ^*d*^ refers to the youngest child in the household.

Parents who had been vaccinated in the past year, particularly those who had received influenza vaccination, had significantly lower hesitancy scores (all *P* < 0.001). Similarly, previous child influenza vaccination was associated with lower hesitancy scores (28.53 ± 17.07 vs 39.29 ± 19.03, *P* < 0.001). A graded inverse association was also observed for vaccination practice scores. Parents with low practice scores (0–2) had the highest hesitancy prevalence (31.05%) and mean hesitancy score (39.31 ± 19.01), whereas those with high practice scores (5–6) had the lowest hesitancy prevalence (6.05%) and mean score (21.30 ± 15.68) (*F* = 842.60, *P* < 0.001; Table 4).

### 3.4. Factors associated with parental hesitancy toward childhood SIV

Univariate analyses of factors associated with parental vaccine hesitancy are presented in Table 4. In multivariable linear and logistic regression analysis (Table 5), older parental age (≥35 years), Shenzhen household registration, employment in enterprises/public institutions, employment in migrant/manual labor occupations, middle-level household income (RMB 10,000–30,000), and kindergarten attendance of the child were independently associated with lower hesitancy scores. In contrast, non-Han ethnicity, having a child aged 3–5 years, and a history of the child being vaccinated in other cities (either exclusively or in addition to Shenzhen) were associated with higher hesitancy scores. With regard to information sources, obtaining vaccine knowledge from online media, kindergartens, and relatives/friends remained independently associated with lower hesitancy scores, whereas the association for healthcare workers was attenuated after adjustment. However, several variables including non-Han ethnicity, employment in migrant/manual labor occupations, having a child aged 3–5 years, obtaining vaccine knowledge from online media, and a history of the child being vaccinated in other cities (either exclusively or in addition to Shenzhen) were no longer significant after dichotomization of the hesitancy scores in logistic regression analysis.

**Table 5:** Multivariable linear and logistic regression analyses of factors associated with parental hesitancy toward seasonal influenza vaccination among preschool-aged children in Shenzhen, China.

Characteristics	N (%)	Multivariate linear regression			Multivariable logistic regression		
		*β*	95% *CI*	*P*	*aOR*	95% *CI*	*P*
Age (years)							
< 35	9624 (53.60)	0.00			1.00		
≥ 35	8331 (46.40)	−1.27	−1.77 ~ −0.77	<0.001	0.87	0.80 ~ 0.95	0.002
Ethnic group							
Han	17201 (95.80)	0.00			1.00		
Others ^*a*^	754 (4.20)	2.05	0.86 ~ 3.24	0.001	1.12	0.92 ~ 1.37	0.255
Marital status							
Married	17482 (97.37)	0.00			1.00		
Others ^*b*^	473 (2.63)	0.18	−1.31 ~ 1.67	0.816	1.05	0.83 ~ 1.33	0.678
Household registration							
Other areas	8121 (45.23)	0.00			1.00		
Shenzhen	9834 (54.77)	−0.97	−1.55 ~ −0.39	0.001	0.80	0.72 ~ 0.89	<0.001
Education level							
High school or lower	5408 (30.12)	0.00			1.00		
Bachelor’s degree or above	12547 (69.88)	0.29	−0.36 ~ 0.94	0.381	1.06	0.95 ~ 1.19	0.263
Occupation							
Commercial and service sectors	3460 (19.27)	0.00			1.00		
Migrant or manual laborers	1169 (6.51)	−1.51	−2.60 ~ −0.42	0.007	0.85	0.71 ~ 1.02	0.086
Employees of enterprises or public institutions	9763 (54.37)	−1.17	−1.82 ~ −0.52	<0.001	0.88	0.79 ~ 0.98	0.026
Unemployed or others	3563 (19.84)	0.26	−0.51 ~ 1.03	0.508	1.05	0.93 ~ 1.19	0.455
Monthly family income (RMB) ^*c*^							
< 10,000	6120 (34.09)	0.00			1.00		
10,000-30,000	7939 (44.21)	−0.83	−1.40 ~ −0.26	0.005	0.83	0.73 ~ 0.93	0.001
≥ 30,000	3896 (21.7)	−0.15	−0.86 ~ 0.56	0.670	0.92	0.71 ~ 1.13	0.137
Number of children							
1	13911 (77.48)	0.00			1.00		
2	3796 (21.14)	−0.05	−0.81 ~ 0.71	0.909	0.93	0.82 ~ 1.06	0.261
≥ 3	248 (1.38)	0.42	−1.71 ~ 2.55	0.699	1.12	0.60 ~ 1.64	0.403
Child’s age (years) ^*d*^							
< 3	3069 (17.09)	0.00			1.00		
3-5	14886 (82.91)	0.88	0.03 ~ 1.73	0.043	1.04	0.91 ~ 1.21	0.547
Child’s sex ^*d*^							
Girl	8091 (45.06)	0.00			1.00		
Boy	9864 (54.94)	0.42	−0.16 ~ 0.79	0.087	1.03	0.95 ~ 1.12	0.448
Whether the child attends kindergarten ^*d*^							
No	1010 (5.63)	0.00			1.00		
Yes	16945 (94.37)	−1.47	−2.52 ~ −0.42	0.006	0.85	0.72 ~ 0.98	0.042
City of previous vaccination of the child							
Only Shenzhen city	10334 (57.55)	0.00			1.00		
Both Shenzhen city and other cities	6599 (36.75)	0.55	0.03 ~ 1.07	0.039	1.09	0.99 ~ 1.19	0.062
Only other cities	1022 (5.69)	1.73	0.68 ~ 2.78	<0.001	1.08	0.90 ~ 1.29	0.402
Acquisition of vaccine knowledge from healthcare workers							
No	4795 (26.71)	0.00			1.00		
Yes	13160 (73.29)	−0.24	−0.78 ~ 0.30	0.389	0.95	0.87 ~ 1.04	0.284
Acquisition of vaccine knowledge from online media							
No	12338 (68.72)	0.00			1.00		
Yes	5617 (31.28)	−0.95	−1.50 ~ −0.40	0.001	0.95	0.85 ~ 1.06	0.901
Acquisition of vaccine knowledge from kindergartens							
No	8579 (47.78)	0.00			1.00		
Yes	9376 (52.22)	−1.20	−1.69 ~ −0.71	<0.001	0.75	0.69 ~ 0.81	<0.001
Acquisition of vaccine knowledge from relatives and friends							
No	14886 (82.91)	0.00			1.00		
Yes	3069 (17.09)	−0.72	−1.39 ~ −0.05	0.035	0.86	0.76 ~ 0.97	0.017
Parents’ knowledge scores on influenza and influenza vaccines							
0-2	1056 (5.89)	0.00			1.00		
3-4	4599 (25.61)	−6.28	−7.37 ~ −5.19	<0.001	0.51	0.44 ~ 0.58	<0.001
5-6	12300 (68.50)	−19.98	−21.03 ~ −18.92	<0.001	0.11	0.10 ~ 0.13	<0.001
Parents’ practice scores for vaccination							
0-2	5925 (33.00)	0.00			1.00		
3-4	10641 (59.26)	−6.18	−6.71 ~ −5.65	<0.001	0.51	0.47 ~ 0.56	<0.001
5-6	1389 (7.74)	−12.61	−13.58 ~ −11.63	<0.001	0.23	0.18 ~ 0.29	<0.001

Note: Others ^*a*^, ethnic minority groups; Others ^*b*^, unmarried, divorced, or widowed; RMB ^*c*^, Chinese yuan; ^*d*^ refers to the youngest child in the household.

Strong graded inverse associations were observed for both knowledge and practice scores. Compared with parents with knowledge scores of 0–2, those with scores of 3–4 and 5–6 had significantly lower hesitancy scores (*β* = −6.28, 95% *CI*: −7.37 to −5.19, *P* < 0.001; *β* = −19.98, 95% *CI*: −21.03 to −18.92, *P* < 0.001, respectively). Similar graded inverse associations were observed in the logistic regression model, with aORs of 0.51 (95% *CI*: 0.44–0.58) and 0.11 (95% *CI*: 0.10–0.13), respectively. Likewise, parents with practice scores of 3–4 and 5–6 had significantly lower hesitancy scores than those with scores of 0–2 (*β* = −6.18, 95% *CI*: – 6.71 to −5.65, *P* < 0.001; *β* = −12.61, 95% *CI*: −13.58 to −11.63, *P* < 0.001, respectively), Correspondingly, the odds of vaccine hesitancy were significantly lower among parents with higher practice scores (aOR = 0.51, 95% *CI*: 0.47–0.56; aOR = 0.23, 95% *CI*: 0.18–0.29, respectively), indicating graded inverse associations between knowledge/practice scores and vaccine hesitancy.

### 3.5. Sensitivity analyses

Sensitivity analyses excluding the two behavioral PACV items (Q1 and Q2) yielded generally similar findings (S1 Table). Higher knowledge and vaccination practice scores remained significantly associated with lower hesitancy scores after exclusion of the behavioral items. Although several effect estimates were slightly attenuated, the overall direction and statistical significance of the main associations remained largely consistent, supporting the robustness of the primary analyses.

## 4. Discussion

In this large cross-sectional survey of 17,955 parents in Shenzhen, 19.42% were classified as hesitant toward SIV for preschool-aged children. This prevalence is lower than that reported in several previous studies from other regions of China [24,29–33]. One possible explanation is that Shenzhen has implemented publicly funded influenza vaccination programs for school-aged children for several consecutive years [22,23], which may have increased public awareness of influenza and vaccination. This heightened awareness was evidenced by the generally high level of parental knowledge regarding influenza and influenza vaccination observed in our sample, although notable gaps remained regarding the need for annual vaccination and the optimal vaccination window. Differences in survey timing, population composition, and measurement tools may also contribute to variation in reported hesitancy rates [34–36].

Although 19.42% of parents were classified as vaccine hesitant, only 19.16% reported that their child had received influenza vaccination in the past year. These closely aligned but conceptually distinct findings suggest that low coverage cannot be explained solely by hesitancy. Policy-related barriers, such as out-of-pocket payment, the exclusion of SIV from the NIP, and insufficient parental awareness regarding the need for annual influenza vaccination, may also constrain uptake. In settings where childhood influenza vaccination is publicly funded, substantially higher vaccination coverage has been reported [17–19]. Given the relatively low influenza vaccination uptake observed in this study, extending Shenzhen’s publicly funded influenza vaccination program to preschool-aged children may help improve vaccine coverage and reduce influenza-related disease burden in this high-risk population. Accordingly, efforts to improve childhood SIV uptake should address both demand-side barriers (e.g., vaccine hesitancy) and system-level barriers (e.g., vaccine financing and accessibility).

In the present study, parental hesitancy toward SIV appeared to reflect not only concerns about vaccine safety, but also doubts regarding vaccine effectiveness and the overall perceived balance between vaccination risks and benefits. Although concerns about serious adverse reactions were the most common hesitant response, more than half of parents also questioned whether the influenza vaccine could effectively protect their children. These findings suggest that parental decision-making may involve weighing the perceived benefits of vaccination against potential risks, rather than focusing on safety concerns alone [11]. Given that trust in healthcare providers and official institutions was relatively high in this study, future communication strategies should move beyond simple safety reassurance. In addition to addressing concerns about adverse reactions, evidence-based communication should also explain why annual influenza vaccination is recommended, how vaccine effectiveness varies across influenza seasons, and how vaccination can still reduce disease severity and complications even when protection is incomplete. Such approaches may help parents make more balanced risk-benefit assessments regarding influenza vaccination.

In the present study, vaccine hesitancy scores differed significantly between parents with correct and incorrect knowledge regarding influenza and influenza vaccination, as well as between those with and without positive vaccination-related behaviors. After categorizing knowledge and practice scores into three levels, higher score categories were independently associated with lower hesitancy scores in multivariable linear regression analyses and lower odds of vaccine hesitancy in multivariable logistic regression analyses, respectively, demonstrating consistent inverse graded associations. These findings are consistent with previous studies reporting that better understanding of influenza and influenza vaccines, as well as positive prior vaccination experiences, are associated with greater parental acceptance of influenza vaccination [11,37]. However, because of the cross-sectional design, the temporal direction of these associations cannot be determined, and parents with lower hesitancy may also be more likely to seek and retain vaccine-related information or engage in positive vaccination practices. In addition, some conceptual overlap exists between the vaccination practice score and the behavioral domain of the adapted PACV scale, which may have partially inflated the observed associations. Nevertheless, sensitivity analyses excluding the behavioral PACV items yielded generally consistent findings, supporting the robustness of the primary results.

Several sociodemographic and vaccination-related variables remained significantly associated with continuous hesitancy scores in multivariable linear regression analyses but were no longer statistically significant after dichotomization of the PACV scores in logistic regression analyses. This finding suggests that factors such as non-Han ethnicity, migrant/manual labor occupations, having a child aged 3–5 years, obtaining vaccine information from online media, and a history of vaccination in other cities may influence the degree of parental vaccine hesitancy without necessarily shifting parents across the predefined threshold used to classify vaccine hesitancy. The attenuation of statistical significance in logistic regression analyses may also reflect information loss resulting from dichotomization of a continuous hesitancy scale.

In contrast, several factors remained consistently associated with lower vaccine hesitancy across both regression models, including older parental age, Shenzhen household registration, parents employed in enterprises/public institutions, middle-level household income, kindergarten attendance, obtaining vaccine information from kindergartens or relatives and friends, and higher parental knowledge and vaccination practice scores. These findings suggest that vaccine hesitancy may be associated not only with individual knowledge and prior vaccination experiences, but also with broader social and structural factors, including social support networks, institutional connectedness, and access to health-related information. Different vaccine information sources also showed distinct associations with parental vaccine hesitancy after adjustment. Vaccine information obtained from kindergartens and from relatives or friends remained significantly associated with lower hesitancy, whereas information obtained from healthcare workers was no longer independently associated with hesitancy. One possible explanation is that communication delivered through kindergartens and social networks may involve more frequent, context-specific, and experience-based interactions that are more readily incorporated into parental decision-making. In contrast, communication from healthcare workers may often occur during brief clinical encounters or after vaccination decisions have largely been formed, thereby limiting its influence on hesitant parents. In addition, vaccine-related information provided by healthcare workers may be more technical and less tailored to parents’ practical concerns regarding influenza vaccination. Notably, the consistent associations observed for kindergarten attendance and kindergarten-based vaccine information highlight the potential value of kindergartens as important platforms for influenza vaccination education and promotion among parents of preschool-aged children.

This study has several strengths, including the large sample size, multistage sampling across six districts, and use of a widely used hesitancy instrument with good overall internal consistency. Several limitations should also be acknowledged. Firstly, vaccination behaviors were self-reported and may be subject to recall or social desirability bias. Secondly, because this study was conducted in Shenzhen, a highly developed urban setting, the findings may not be fully generalizable to less-developed regions in China. However, 45.23% of the participants did not hold Shenzhen household registration, suggesting a relatively diverse study population and providing some degree of external relevance to other urban areas with substantial migrant populations. Moreover, potential selection bias should be considered, such as although the six selected districts covered Shenzhen’s major population and economic areas, they did not include all administrative districts. And parents recruited from community health service centers or kindergartens/childcare institutions may have had greater health awareness than those not reached through these settings. Thirdly, use of an online questionnaire may have introduced selection bias, because parents with greater digital literacy or health awareness may have been more likely to participate. Fourthly, the cross-sectional design precludes any causal inference, and the term “graded association” is used purely descriptively. Fifthly, the cut-off points used to categorize knowledge and practice scores were not based on prior validation studies and should therefore be interpreted cautiously. These categorizations were primarily used to facilitate interpretation of graded associations and may not represent clinically meaningful thresholds. Finally, the adapted PACV instrument showed only moderate internal consistency in some subdomains, indicating that further psychometric validation for SIV-specific use in this population may be warranted.

## 5. Conclusion

In conclusion, parental hesitancy toward SIV among preschool-aged children in Shenzhen was low-to-moderate and was associated with concerns regarding both vaccine safety and effectiveness. Higher parental knowledge and more favorable prior vaccination practices were consistently associated with lower vaccine hesitancy. Interventions that improve influenza and vaccine-related knowledge, address parental concerns regarding vaccine safety and effectiveness, and strengthen communication through trusted community-based channels such as kindergartens may help reduce vaccine hesitancy. In parallel, policies aimed at improving the affordability and accessibility of childhood influenza vaccination, including consideration of publicly funded vaccination programs for preschool-aged children, may help improve vaccine uptake.

## Supporting information

S1 Dataset Dataset underlying the analysis of parental knowledge, vaccine hesitancy, and vaccination practices regarding seasonal influenza vaccination for preschool-aged children in Shenzhen, China. This file contains the de-identified dataset used for the analyses reported in this study.
(XLSX)

S1 Table Sensitivity analyses of multivariable linear regression models using revised PACV scores excluding the two behavioral items (Q1 and Q2). (DOCX)

## Abbreviations

NIP: National Immunization Program
SIV: seasonal influenza vaccination
PACV: parental attitudes toward childhood vaccines
CI: confidence interval
KMO: Kaiser–Meyer–Olkin
QR: quick response
RMB: Renminbi
SD: standard deviation
VIF: variance inflation factor
