# Supplementary material for: Parental knowledge, vaccine hesitancy, and practices regarding seasonal influenza vaccination for preschool-aged children in Shenzhen, China: Insights from a cross-sectional survey
Source: PLoS One. 2026 Jul 9;21(7):e0353478. doi: 10.1371/journal.pone.0353478 (PMC13349090; doi:10.1371/journal.pone.0353478)
Supplement: S1 Table — (DOCX) [file pone.0353478.s002.docx]

**S1 Table**

Sensitivity analyses of multivariable linear regression models using revised PACV scores excluding the two behavioral items (Q1 and Q2)

| Characteristics | Multivariate linear regression | | |
| --- | --- | --- | --- |
|  | *β* | 95% *CI* | *P* |
| Age (years) |  |  |  |
| <35 | 0.00 |  |  |
| ≥35 | -1.08 | -1.60~ -0.57 | <0.001 |
| Ethnic group |  |  |  |
| Han | 0.00 |  |  |
| Others *^a^* | 2.43 | 1.20~ 3.66 | <0.001 |
| Marital status |  |  |  |
| Married | 0.00 |  |  |
| Others *^b^* | 0.03 | -1.57~ 1.63 | 0.967 |
| Household registration |  |  |  |
| Other areas | 0.00 |  |  |
| Shenzhen | -0.78 | -1.39~ -0.18 | 0.011 |
| Education level |  |  |  |
| High school or lower | 0.00 |  |  |
| Bachelor's degree or above | 0.32 | -0.35~0.99 | 0.352 |
| Occupation |  |  |  |
| Commercial and service sectors | 0.00 |  |  |
| Migrant or manual laborers | -1.52 | -2.65 ~ -0.39 | 0.008 |
| Employees of enterprises or public institutions | -0.91 | -1.59~ -0.23 | 0.008 |
| Unemployed or others | 0.20 | -0.60 ~ 1.00 | 0.630 |
| Monthly family income (RMB) *^c^* |  |  |  |
| <10,000 | 0.00 |  |  |
| 10,000-30,000 | -0.50 | -1.09 ~ 0.09 | 0.097 |
| ≥30,000 | -0.18 | -0.92 ~ 0.55 | 0.623 |
| Number of children |  |  |  |
| 1 | 0.00 |  |  |
| 2 | -0.25 | -1.04 ~ 0.54 | 0.537 |
| ≥3 | -0.18 | -2.38 ~ 2.03 | 0.875 |
| Child’s age (years) *^d^* |  |  |  |
| <3 | 0.00 |  |  |
| 3-5 | 0.85 | -0.03 ~ 1.73 | 0.059 |
| Child’s sex *^d^* |  |  |  |
| Girl | 0.00 |  |  |
| Boy | 0.22 | -0.27 ~ 0.71 | 0.380 |
| Whether the child attends kindergarten *^d^* |  |  |  |
| No | 0.00 |  |  |
| Yes | -1.32 | -2.40 ~ -0.24 | 0.017 |
| City of previous vaccination of the child |  |  |  |
| Only Shenzhen city | 0.00 |  |  |
| Both Shenzhen city and other cities | 0.42 | -0.12 ~ 0.95 | 0.131 |
| Only other cities | 1.17 | 0.09 ~ 2.25 | 0.035 |
| Acquisition of vaccine knowledge from healthcare workers |  |  |  |
| No | 0.00 |  |  |
| Yes | -0.09 | -0.65 ~ 0.47 | 0.753 |
| Acquisition of vaccine knowledge from online media |  |  |  |
| No | 0.00 |  |  |
| Yes | -1.26 | -1.83 ~ -0.70 | <0.001 |
| Acquisition of vaccine knowledge from kindergartens |  |  |  |
| No | 0.00 |  |  |
| Yes | -1.11 | -1.61 ~ -0.60 | <0.001 |
| Acquisition of vaccine knowledge from relatives and friends |  |  |  |
| No | 0.00 |  |  |
| Yes | -1.14 | -1.83 ~ 0.50 | 0.001 |
| Parents’ knowledge scores on influenza and influenza vaccines |  |  |  |
| 0-2 | 0.00 |  |  |
| 3-4 | -5.92 | -7.05 ~ -4.80 | <0.001 |
| 5-6 | -20.19 | -21.27 ~ -19.10 | <0.001 |
| Parents’ practice scores for vaccination |  |  |  |
| 0-2 | 0.00 |  |  |
| 3-4 | -6.82 | -7.37 ~ -6.27 | <0.001 |
| 5-6 | -13.85 | -14.86 ~ -12.85 | <0.001 |

Note: Others *^a^*, ethnic minority groups; Others *^b^*, unmarried, divorced, or widowed; RMB *^c^*, Chinese yuan; *^d^* refers to the youngest child in the household.
